# Metagenomic Profiling and Microbial Metabolic Potential of Perdido Fold Belt (NW) and Campeche Knolls (SE) in the Gulf of Mexico

**DOI:** 10.3389/fmicb.2020.01825

**Published:** 2020-08-11

**Authors:** Luciana Raggi, Fernando García-Guevara, E. Ernestina Godoy-Lozano, Adrian Martínez-Santana, Alejandra Escobar-Zepeda, Rosa María Gutierrez-Rios, Antonio Loza, Enrique Merino, Alejandro Sanchez-Flores, Alexei Licea-Navarro, Liliana Pardo-Lopez, Lorenzo Segovia, Katy Juarez

**Affiliations:** ^1^Instituto de Biotecnología, Universidad Nacional Autónoma de México, Cuernavaca, Mexico; ^2^CONACYT-Laboratorio de Biotecnología Acuícola, Instituto de Investigaciones Agropecuarias y Forestales, Universidad Michoacana de San Nicolás de Hidalgo, Morelia, Mexico; ^3^Centro de Investigación Sobre Enfermedades Infecciosas, Departamento de Bioinformática en Enfermedades Infecciosas, Instituto Nacional de Salud Pública, Cuernavaca, Mexico; ^4^Laboratorio de Inmunología Molecular y Biotoxinas, Departamento de Innovación Biomedica, CICESE, Ensenada, Mexico

**Keywords:** Gulf of Mexico, bacterial community structure, metagenome, metabolic potential, hydrocarbon degradation

## Abstract

The Gulf of Mexico (GoM) is a particular environment that is continuously exposed to hydrocarbon compounds that may influence the microbial community composition. We carried out a metagenomic assessment of the bacterial community to get an overall view of this geographical zone. We analyzed both taxonomic and metabolic markers profiles to explain how the indigenous GoM microorganims participate in the biogeochemical cycling. Two geographically distant regions in the GoM, one in the north-west (NW) and one in the south-east (SE) of the GoM were analyzed and showed differences in their microbial composition and metabolic potential. These differences provide evidence the delicate equilibrium that sustains microbial communities and biogeochemical cycles. Based on the taxonomy and gene groups, the NW are more oxic sediments than SE ones, which have anaerobic conditions. Both water and sediments show the expected sulfur, nitrogen, and hydrocarbon metabolism genes, with particularly high diversity of the hydrocarbon-degrading ones. Accordingly, many of the assigned genera were associated with hydrocarbon degradation processes, *Nitrospira* and *Sva0081* were the most abundant in sediments, while *Vibrio*, *Alteromonas*, and *Alcanivorax* were mostly detected in water samples. This basal-state analysis presents the GoM as a potential source of aerobic and anaerobic hydrocarbon degradation genes important for the ecological dynamics of hydrocarbons and the potential use for water and sediment bioremediation processes.

## Introduction

The Gulf of Mexico (GoM) is a basin with one of the most significant oil reservoirs in the world. There are few microbial diversity studies in Mexican waters of the GoM (Lizarrága-Partida, 1986; Rosano-Hernández et al., 2012; Sanchez-Soto et al., 2018; Ramírez et al., 2020) in contrast to an extensive effort along the northeastern coast, particularly related to the Deep Water Horizon oil spill (Kostka et al., 2011; Liu et al., 2012; Mason et al., 2014). Worldwide, oceans’ microbiology has been studied principally throughout the water-column and hot-spots (i.e., seeps, hydrothermal vents, oil discharge sites, etc.). There are very few metagenomic studies from typical “desert-like” sediments, as the latest endeavors to cover all the oceans (Zinger et al., 2011; Bienhold et al., 2016), and whole Earth (Thompson et al., 2017).

The way to study, analyze, interpret, and present microbial diversity data is still quite complex. With the popularization of metagenomics with new generation sequencing techniques, hundreds of studies are carried out yearly, and several methods to report microbial diversity are currently in use. Computational strategies are being developed to try to disentangle microbial richness and functional data, the most used nowadays is statistical correlation analyses. However, network-based modeling methods and the integration of several distinct approaches, could permit a more comprehensive understanding of microbial ecology (Chong and Xia, 2017). The analysis or simulation of functional traits linked directly to microbial richness has seldom been done. Commonly, microbial taxon structure (microbial community structure) and functional traits or interactions (functional dynamics) are analyzed independently. One of the first efforts to integrate richness and function is shown in Delmont et al. (2018); where the importance of *Planctomycetes* within the essential nitrogen-fixing microbial population through both a phylogenetic and a functional network analysis was demonstrated.

Biogeochemical cycles in sediments, water and air are influenced by microbial metabolism. Also, climate, currents, rivers, faults, hydrocarbon emanations, among other natural processes, and pollution or other anthropogenic activities, change or disrupt microbial profiles and therefore their role in the cycles. The GoM is known for having different depositional environments in which the accumulation of sediments ranges widely (Davis, 2017). In many of these locations, microorganisms reduce sulfate ions to hydrogen sulfide, which is an essential energy source for many free living and symbiotic organisms. The sedimentary depositional process known as diagenesis is the source of organic matter that is oxidized through the sulfate reduction process in sediments (Kasten and Jørgensen, 2000).

Genes involved in dissimilatory sulfate reduction (DSR) such as *sat* (sulfate adenylyltransferase), *aprA* (adenylylsulfate reductase), and *dsrAB* [dissimilatory (bi) sulfate reductase], a key enzyme in the biogeochemical sulfur cycle, have been used as marker genes for phylogenetic affiliation of the different taxonomic groups that participate in the sulfur cycle. Although sulfate oxidation lacks a preserved metabolic pathway like DSR, *soxB* (*S*-sulfosulfanyl-L-cysteine sulfohydrolase) has been used as an excellent functional gene marker for sulfur oxidation in different studies (Meyer and Kuever, 2007a; Hügler et al., 2010; Jaffer et al., 2019), and *aprA* and *dsrA* have previously been used as functional maker genes for sulfate-reducing but also sulfur-oxidizing microorganisms (Meyer and Kuever, 2007b).

Markers for nitrate reduction, denitrification and dissimilatory nitrate reduction to ammonium (DNRA) have been used to study the community diversity of environmental samples genes (Braker et al., 2000). *narG* gene codes for membrane-bound nitrate reductase, *napA* gene for periplasmic nitrate reductase, both catalyzing the reduction of nitrate to nitrite (Moreno-Vivián et al., 1999). *nirS, nirK* (haem-cytochrome cd-1 type and copper-containing type nitrite reductases), *norB* (nitric oxide reductase) and *nosZ* (nitrous oxide reductase), are four markers that have been used to detect communities of denitrifying bacteria (Braker et al., 1998; Smith et al., 2007). *nrfA* gene has been used as a marker for nitrite reductase in DNRA, catalyzing the six-electron reduction of nitrite to ammonium (Stach et al., 2000; Klotz et al., 2008; Kern et al., 2011), coupled to oxidation of electron donors, such as organic matter, ferrous iron, hydrogen, sulfide, and methane (Burgin and Hamilton, 2007). Anammox bacteria extract energy by producing molecular nitrogen from ammonium and nitric oxide. Previous studies searching for environmental diversity of anammox bacteria employed the genes *hzsA* coding for hydrazine synthase (Harhangi et al., 2012) and the gene *hdh* or *hdo* coding for hydrazine dehydrogenase (Kong et al., 2013).

GoM is exposed to a continuous hydrocarbon flux through natural seeps, independently of oil-spills, therefore, its bacterial community must be adapted to oil and degradation processes. Oil from natural leaks concentrate has been regionally concentrated in the GoM, and 68, 25, 7, and <1% of the total has been detected in the NW, SW, NE, and SE gulf regions, respectively (Macdonald et al., 2015). Hydrocarbon degraders are active and blooming whenever an oil-spill occurs, then hydrocarbon degradation genes must be present within indigenous bacterial communities. The first step, in both aerobic and anaerobic hydrocarbon degradation, involves chemical activation, and subsequently, the compound is channeled into central metabolism (Fuchs et al., 2011; Lueders and Von Netzer, 2014). To explore hydrocarbon degradation gene families associated to the activation in aerobic degradation of alkanes have been used as are the alkane hydroxylases *alkB* and CYP153 (Nie et al., 2014), and for aerobic degradation of aromatic compounds the Rieske non-heme iron dependent oxygenases (Duarte et al., 2014). For the anaerobic degradation of hydrocarbons *bssA* benzyl succinate like synthases (Callaghan et al., 2010) between others have been used as markers.

We have previously reported the microbial diversity found in southwestern GoM sediments (Godoy-Lozano et al., 2018). In this study, we continue to investigate this geographical zone, in particular the Campeche Knolls, and also the Perdido Fold Belt region off the coast of Tamaulipas (NW). In these two areas, natural oil seeps have been detected (Macdonald et al., 2015) and particular oil-adapted bacterial communities are expected. We carried out a metagenomic assessment of the bacterial community in water column and sediment, and searched for metabolic marker genes of carbon, nitrogen, sulfur and hydrocarbon metabolism to set these GoM regions in a biogeochemical context.

## Materials and Methods

### Sample Collection and Processing

**FIGURE 1:**
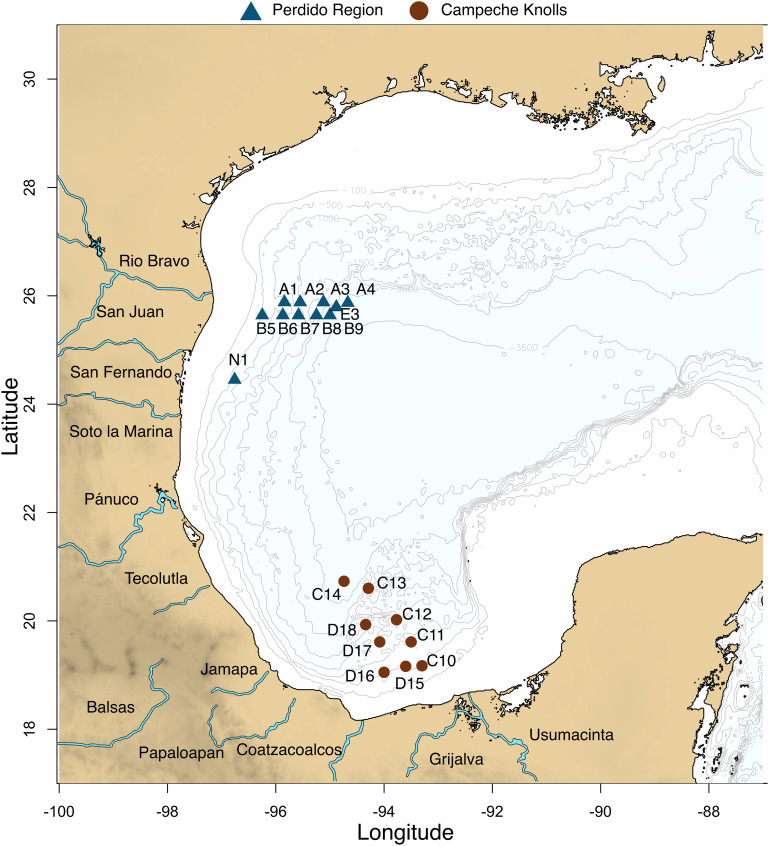
Twenty locations were sampled in Mexican waters from the Gulf of Mexico. Blue triangles represent Perdido region and brown circles Campeche Knolls. Nineteen samples were collected in March 2016. An extra sample (E03) in September 2015.

Water and sediments from 19 locations at the GoM were collected in March 2016. Two longitudinal transects corresponding to the Perdido region, and two latitudinal transects corresponding to a southern region in Campeche Knolls (Figure 1 and Supplementary Table S2) were sampled. Seawater was collected using a rosette with 20-l Niskin bottles at four depths: maximum fluorescence (MAX), minimum oxygen zone (MIN), 1,000 m depth at the Antarctic Intermediate Water (AAIW), that assures a good comparison because it is a well-oxygenated water, and bottom water (DEEP), that varied between 550 and 3,200 m depth. A total of 57 water samples were collected and analyzed: 14 MAX, 17 MIN, 9 of AAIW (1,000 m), and 17 DEEP samples. Each seawater 100 L sample was concentrated to approximately 3 L with a tangential filtration system (KrosFlo; mean/rating: mPES7100 kD; surface area: 2.55 m^2^). From this volume, 100 mL were stored at 4°C for filtration through a Sterivex filter or passed through a 0.22 μm polycarbonate membrane (Merck Millipore). Immediately afterward, the filters were stored in liquid N_2_. Sediment was brought to the ship surface with a box corer, and then with a manual tube core, a sample of the first 10 cm was collected (SED). An extra sample (E03) was taken in a pre-expedition in September 2015. All samples were kept frozen at −20°C until processed.

### Physicochemical Parameters

Oxygen, fluorescence, temperature and salinity were measured along the water column with a CTD (SBE-9) coupled to the rosette, and sampling was determined based on these parameters. Oxygen values were between 7.07 and 3.6 mg/L at the Perdido Fold Belt zone, and from 6.9 to 3.5 mg/L in the Campeche Knolls area. The lowest oxygen levels detected were between 320 and 450 m depth (MIN samples). Fluorescence values were between 0.07 and 1.22, being the highest between 50 m and 70 m depth (MAX samples). Temperatures were between 21.7 to 4.3°C and salinity between 34.9 and 36.3 in the Perdido zone and 24.86 to 4.38°C and 34.9 to 36.1 salinity in the Campeche area (Supplementary Table S1). Due to the lack of instrumentation, parameters of the sediments were not measured, we can only extrapolate from measurements performed in Godoy-Lozano et al. (2018) sampling.

### Metagenomic DNA Extraction and Sequencing

This study is immersed in a major Mexican project which included studying the diversity of the Gulf of Mexico^1^. Our goal here was to describe and study the bacterial community. Our choice would have been to cover all sites with shotgun analysis, however, we could not, due to economic constraints and therefore we covered all sites with amplicon metagenomics. Although primers to amplify both bacteria and archaea or simply primers to get the most archaea (Klindworth et al., 2013) are available, we standardized the use of primers throughout the CIGOM consortium, as well as DNA extraction.

DNA extraction was performed from Sterivex or Millipore water samples filters using the Power Water DNA isolation kit (MO BIO-QIAGEN), or from 0.5 g of surface sediment, using the PowerSoil DNA Isolation Kit (MO BIO-QIAGEN). Extracted metagenomic DNA was used to prepare libraries for Illumina sequencing of variable V3–V4 regions from 16S rRNA taxonomic marker gene. Amplification of the V3–V4 region was carried out with primer pair S-D-Bact-0341-b-S-17 and S-D-Bact-0785-a-A-21 (Klindworth et al., 2013), and PCR conditions were 98°-3 min/98°-3 s/50°-1.5 min/72°-1 min/72°-10 min, for 25 cycles with Phusion Polymerase (Thermo Scientific). For library preparation, the Nextera XT DNA Library Prep Kit (Illumina) protocol was used following the Illumina’s protocol. Libraries sequencing was accomplished in an Illumina MiSeq platform with a paired-end reads configuration of 300 cycles by side. Amplicon raw reads are available at the SRA site under project id PRJNA609564 (SAMN14259149-SAMN14259166, SAMN14255300-SAMN14255357), and PRJNA380945 (SAMN06649846).

Additionally, water and sediment samples with the better DNA extraction yield (D18_MAX, A04_MIN, A04_AAIW, A04_SED, E03_SED, D18_SED) were used to perform whole metagenome shotgun sequencing. These libraries were prepared using the TruSeq DNA PCR-Free HT Library Prep (Illumina) kit. Sequencing of paired-end reads was carried out in an Illumina NextSeq500 platform with a configuration of 75 or 150 cycles by side (see Supplementary Table S2. Sample stats, “WMS” sheet). Metagenomic reads were also submitted to SRA under the projects PRJNA609564 (SAMN14329496, SAMN14329497, and SAMN14329491-SAMN14329493) and PRJNA380945 (SAMN06649846).

### Bioinformatic Analysis: Amplicon Profiles and Taxonomic Assignment, and Whole Metagenome Shotgun (WMS) Data

16S rRNA amplicon samples were sequenced to obtain a yield of 1-5 e + 05 paired-end reads, from which, the overlapping reads ratio was ∼70–94% (see Supplementary Table S2) using the Flash v1.2.7 software (Magoč and Salzberg, 2011). Merged reads were used for both OTUs clustering and taxonomic annotation analysis. For taxonomic annotation the extended fragments were denoised using DADA2 algorithm with the R Package dada2 (Callahan et al., 2016). Afterward, ASVs (amplicon sequence variants) reads were annotated for taxonomy using the Sklearn tool within the QIIME 2 pipeline (Bolyen et al., 2019) against the SILVA 132 SSU database (Quast et al., 2013).

The extended fragments were clustered in OTUs at 97% identity (OTUs_0_._03_) using VSEARCH tool v2.4.3 (Rognes et al., 2016). To discard artificial diversity, we filtered chimeric sequences, and clusters containing one sequence were discarded. Sampling effort was calculated as well as the Chao1 and Shannon alpha diversity indices with R Phyloseq library (Mcmurdie and Holmes, 2013). Finally, the filtered matrix was normalized using the metagenomeSeq v1.16.0 method (Mcmurdie and Holmes, 2014), and a beta diversity distance matrix was calculated using the Bray–Curtis index.

To perform the metabolic potential prediction of samples sequenced by WMS, we assembled genomic fragments from reads using the IDBA-UD v1.1.1 (Peng et al., 2010) metagenomic assembler. Thereafter, we predicted the coding DNA sequences (CDS) from contigs by MetaGeneMark v3.26 (Zhu et al., 2010), and annotated the translated amino acidic chains by blastp 2.2.31+ (Camacho et al., 2009) against the Swissprot database included in the Trinotate v3.0.1 annotation suite (Bryant et al., 2017). Uniprot annotations were retrieved from the Trinotate output. Gene abundance was estimated as coverage through mapping reads to contigs using BWA v0.7.12-r1039 (Li and Durbin, 2009) and coverageBed function from bedtools v2.25.0 (Quinlan and Hall, 2010).

### Networks Analysis

We built a network using the assigned KO number and taxonomic information of the closest Uniprot hit (*e*-value cutoff 1e-5) from the Trinotate annotation report. Nodes in the network represent either a KO or a genus. Edges represent a found KO-genus pair and edge-width is proportional to the log2 of total gene counts found for that pair. We included selected genes (KO numbers) that are markers for photosynthesis and for hydrocarbon degradation, sulfur metabolism, nitrogen metabolism, and methane metabolism pathways in the final network. Networks were generated with the Cytoscape software v.3.6.1 (Shannon et al., 2003). The network heterogeneity values calculated with Cytoscape are included, as they show the topological feature of complex networks with the degree of interactions. A higher value means a more complex network.

### Metabolic Markers Search

WMS analysis revealed important and interesting archaeal genes, thus we decided to include them in the analyses. We searched for homologous sequences of genes related to sulfur (*sat, aprA*, *dsrAB*, *soxB*), nitrogen (*nrfA, nosZ, norB, nirS, nirK, nifH, narG, napA, nxrB, hzsA, hdh, hao, amoA*), methane (*mcrA*, *mer*, *fmd, ftr*, *mch, mtd*, *mtrA*) and hydrocarbon (*acsB*, *alkB*, *bssA-like*, *Fe-Fe_Hydrogenases*, *Ni-Fe_Hydrogenases*, *Rieske_superfamily* genes, *rplC* and *rplE*) metabolism using GraftM v0.11.1 (Boyd et al., 2018) through the metagenome assemblies, using an e-value cutoff of 1e-30. The packages of the genes *sat, mer*, *nosZ*, *nirK*, *nifH*, *narG*, *napA*, *napA*, *nxrB*, *hao*, *acsB*, *Fe-Fe_Hydrogenases*, *Ni-Fe_Hydrogenases*, *rplC* and *rplE* were retrieved from http://github.com/geronimp/graftm_gpkgs/tree/master/7 (Boyd et al., 2018) through the metagenome assemblies, using an e-value cutoff of 1e-30. The gene packages of *aprA*, *soxB*, *fmd*, *ftr*, *mch*, *mtd*, *mtrA*, *nrfA*, *norB*, *nirS*, *hzsA*, and *hdh* were constructed collecting sequences from Uniprot (Uniprot Consortium, 2018) and using their corresponding NCBI taxonomy. Finally, gene packages of the following genes were manually built based on previous studies as reference for taxonomic annotation: *dsrAB* (Müller et al., 2015), *alkB* (Nie et al., 2014), *bssA*-like (Acosta-González et al., 2013), Rieske_superfamily genes (Duarte et al., 2014), and *amoA* which included ammonia-oxidizing archaea (AOA) and ammonia-oxidizing bacteria (AOB) markers, plus *pmoA* (Khadka et al., 2018).

Gene package files constructed in this study can be found at https://github.com/garciafertson/mmf1_gpkgs.git. After homologous sequence identification with GraftM, we calculated the relative abundance for the identified genes in relation to the total number of predicted genes in the sample; thus we extracted each identified sequence and added the sequencing depth value divided by the total number of genes in the metagenome assembly times the metagenome’s average sequencing depth: gene abundance = [sum(detected_gen_sequencing_depth)]/(total_ number_of_genes^∗^total_sequencing_depth).

## Results

### Community Distribution by Habitats and Alpha Diversity of Perdido Fold Belt (NW) and Campeche Knolls (SE)

**FIGURE 2:**
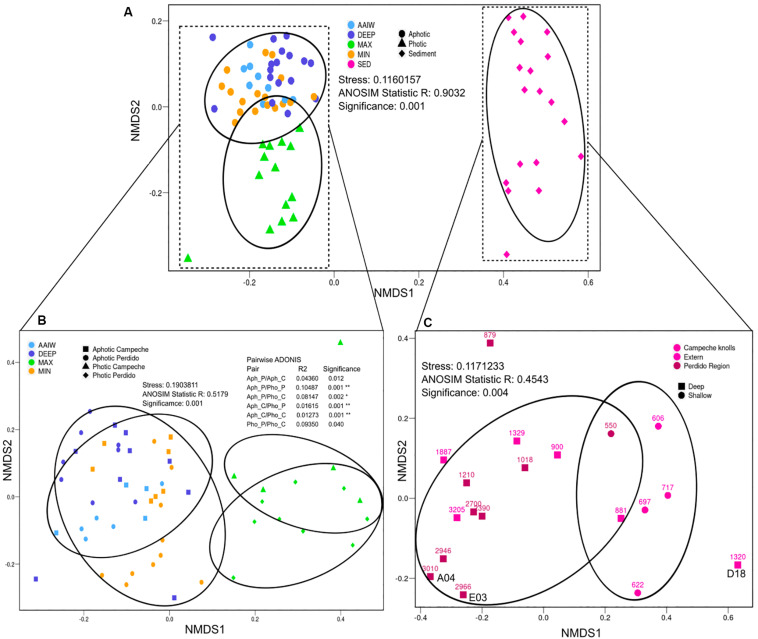
Beta diversity NMDS. Non-metric Multidimensional Scaling plot (NMDS) depicting Bray–Curtis dissimilarity distance for ASVs diversity. **(A)** All samples where labeled according to the sample type (color) and category (shape). **(B)** Water where samples labeled according to the sample type (color), and geographical origin with photic or aphotic category (shape). **(C)** Sediment where samples labeled according to their geographical region (color) and depth (shape), where shallow groups correspond to lower than 800 m. Ellipses represent the area enclosing the group at 90% of confidence. Analysis of ANOSIM and pairwise ADONIS between groups is shown to demonstrate group segregation.

In order to analyze alpha diversity of the different sampling sites, both OTUs_0_._03_ and ASVs were used. The total number of observed OTUs_0_._03_ from both Perdido and Campeche sampling sites varies between 1,589 to 6,170 in the case of the water column samples and from 6,099 to 33,740 in sediment samples. These observed OTUs_0_._03_ represent ∼96% of the maximum OTUs_0_._03_ expected according to the Chao1 index. The Good’s coverage values for these samples are in the range of 0.9096 and 0.9979. We used both ASVs and OTUs to analyze diversity and sample dispersion, however we are aware of the 16S rRNA gene intragenomic heterogeneity which could be reflected in both analysis, with ASVs analysis overestimating, and OTUs clustering at 97% underestimating diversity. However, both calculations were very similar, indicating that both analyses are correct for V3–V4 variable regions. Shannon index for both analyses indicate that sediment samples have greater diversity than water samples, as expected. Within the water samples, the MIN and DEEP samples have higher bacterial diversity than MAX and AAIW (see Supplementary Table S2 for sequencing yields and alpha diversity indices statistics). We generated a Bray–Curtis distance matrix and plotted an NMDS to observe differences in diversity between samples (Figure 2). Sediment samples segregate from the water samples into a single, separated cluster. Among water samples, aphotic layers form a mixed group that includes the MIN, AAIW and DEEP-water layers, and the samples from the MAX layer, form an independent photic cluster (Figure 2A). According to a pairwise ADONIS test, photic and aphotic layers form independent groups that are homogeneous regardless of the geographic origin of the samples (Figure 2B). With respect to sediments we observe a tendency to cluster by water column depth (>800 m or >800 m) independently of their geographical origin, except for sample D18 that does not cluster with any of both groups (Figure 2C).

### Microbial Community Composition of Water Column and Sediments of Perdido Fold Belt (NW) and Campeche Knolls (SE)

#### Water Column

**FIGURE 3:**
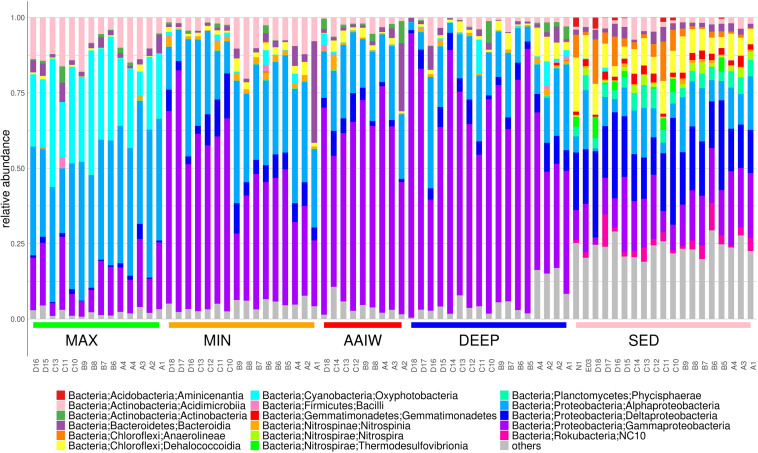
The bar plot displays relative abundances for the 16S rRNA amplicon taxonomic annotations at the Class level. Classes with relative frequencies smaller than 3.5% in every sample were collapsed into the *others* category.

The bacterial community composition is structured by depth as each layer has a characteristic diversity (Figure 3). In the maximum fluorescence layer (MAX), *Oxyphotobacteria* reaches between 8–42% of the relative community abundance, as expected, and with respect to most abundant classes *Alphaproteobacteria* reach 21–49%, *Gammaproteobacteria* 4–24%, *Acidimicrobiia* 4–18% and *Bacteroidia* 0.7–6.4% (Figure 3). In addition to the well-known genus *Synechococcus* that reaches up to 25%, abundant genera related to potential hydrocarbon degrading bacteria (PHDB) such as *Pseudomonas* spp. (6%), *Alcanivorax* (5.4%), and *Alteromonas* (4%) among other less abundant genera, are observed.

The next well-separated layer is MIN (between −253 and −456 m depth), where oxygen levels were 0.5 mg/L lower than in the rest of the whole water column; we observed that *Alphaproteobacteria* decrease 10–40% while *Gammaproteobacteria* increase substantially (21–80%) in the aphotic layers concerning MAX layer and sediments. PHDB are still abundant, at over 1%, as shown by *Halomonas* spp. (5-17%), and *Alcanivorax* spp. (0.1–12%). Other abundant genera at this level are *Sva0996 marine group* that reaches up to 20%, *Methylophaga* 11%, *Roseovarius* 10%, *Leeuwenhoekiella* 32%, *Alteromonas* 27%, and *Synechococcus* CladeIb 11%.

In the next deeper layer, at −1000 m depth (AAIW), *Alphaproteobacteria* decrease even more (15–24%) and *Betaproteobacteria* are not detected. *Halomonas* (1–48%), *Alcanivorax* (0.1–4.3%) *Methylophaga* 12% and *Sva0996 marine group* 6% increase substantially as well as *Vibrio* that reaches 54%. Contrary, *Alteromonas* decrease to 1–13% and *Leeuwenhoekiella* to 22%. *Gammaproteobacteria* continue to increase and top up 43 and 75% in the DEEP layer.

#### Sediments

Bacterial communities in sediment samples are dominated by different proportions of *Deltaproteobacteria* (12–35%), *Gammaproteobacteria* (2–24%), *Alphaproteobacteria* (1–20%) and *Dehalococcoidia* (0.3–17%) (Figure 3). We found that the *Deltaproteobacteria* class is more predominant in deep sediments. Also genera related to sulfur metabolism and hydrocarbon degraders as Wb1-A12 (0.1–10), Urania-1B-19 (1–6.8), *Nitrospira* (0.3–3.5%) *Sva0081 sediment group* (0–4.8). New reclassified *NC10 class* 0.1–10% and *Woeseia* (0.7–6.8%) are also present (Du et al., 2016; Bacosa et al., 2018).

### Linking Taxonomy With Metabolism Through Networks

**FIGURE 4:**
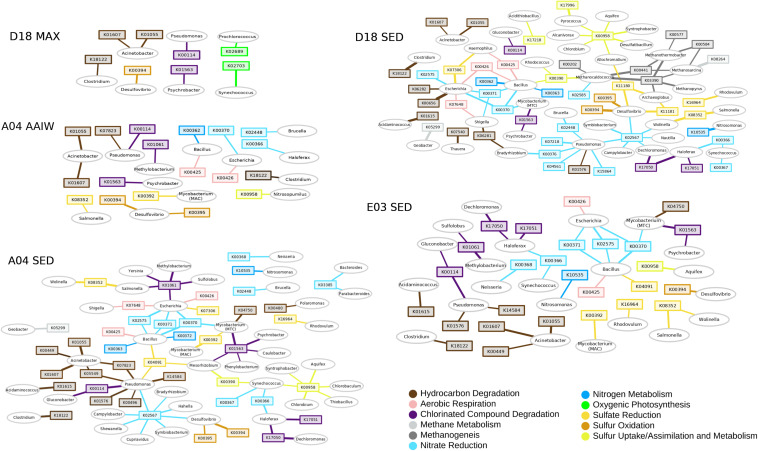
KO and genus networks. Ovals represent genera, squares represent KOs. Edge width is proportional to the logarithm of total counts; only edges and nodes with a total count above 10 are displayed for image clarity. Methane metabolism is shown in light gray and methanogenesis in gray; sulfur oxidation in ocre, sulfate reduction in yellow and sulfur uptake/assimilation in lime; nitrate reduction/denitrification in sky blue and nitrogen metabolism in blue; alkanes/hydrocarbon degradation in dark brown, aromatics degradation in brown and chlorinated compound degradation in purple; oxygenic photosynthesis in green and aerobic respiration in pink (the KO list is included in Supplementary Table S3).

We performed a network analysis to link taxonomy with function (Figure 4). The network heterogeneity values for each shotgun network were: D18MAX = 1.102, A04MIL = 1.302, A04SED = 1.627, D18SED = 1.581, E03SED = 1.430. Bacterial or gene “hubs” are shown in the networks. Each hub should be showing a key-role player in the environment (Banerjee et al., 2018). We discarded genes that are redundant in the metabolism, or that take part in many different metabolic pathways. Some genes and some taxa become a hub, either as an “essential metabolic marker gene” or a “keystone taxon,” respectively. *Pseudomonas* and *Acinetobacter* are detected as bacterial keystone players in all layers. *Synechococcus* and *Prochlorococcus* are keystone players in the MAX layer, as expected. Marker gene K00958 (sulfate adenylyltransferase), a sulfate metabolism gene, is present in all layers as an essential *hub* in sediments. The K02567 module, a marker for denitrification, is also present as a hub in sediments A04 and D18. The D18 sediment (Campeche region) seems to be more metabolically diverse than A04 and E03, showing more genes for alkanes degradation (K00656, K06281, K06282, K07540), and genes for methanogenesis and methane metabolism (K00441, K00577, K0584, K03390, K08264) (Supplementary Table S3). The K01563 gene (haloalkane dehalogenase) is a hub in all three sediments. *Desulfovibrio* is found in all networks, representing a sulfate-reducing bacterial module, and this genus is related to the sulfur metabolism marker genes K00394-K00395 (*aprA-B*, the bidirectional enzyme transforming sulfite to adenylyl sulfate). *Bacillus* and *Escherichia* might represent heterotrophic bacteria, and are associated with respiration and nitrate reduction genes (nitrate reductase *narG* – K00370). *Acinetobacter* and *Pseudomonas* represent the PHDB, and the genes associated belong to the hydrocarbon degradation pathways: K01607 (4-carboxymuconolactone decarboxylase PcaC), K01055 (3-oxoadipate enol-lactonase-PcaD) (Supplementary Table S3).

### Marker Genes Involved in Anaerobic Metabolic Processes in Sediments

#### Sulfur Metabolism

**FIGURE 5:**
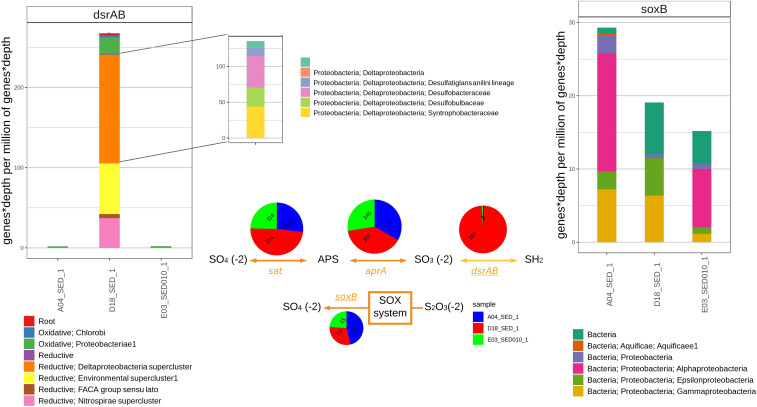
The bar graph on the left displays the abundance of genes with sequence homology to *dsrAB* in shotgun sediment assemblies from A04SED, D18SED, and E03SED samples and their most likely taxonomic classification according to the classification proposed by Müller et al. (2015). Center of the figure shows the relative abundance of genes *aprA, sat, dsrAB* (in the DSR pathway) and *soxB* (thiosulfate oxidation). The bar graph on the right displays the relative abundance of genes with sequence homology to *soxB* in sediment metagenomic assemblies.

The *sat*, *aprA*, and *dsrA* genes, involved in DSR, were detected in the three sediment metagenomes. Their abundance as well as their associated taxonomy show a clear difference between D18 and E03 and A04; genes associated to *Deltaproteobacteria* (mostly *dsrAB*) are more abundant in D18 (it shows up as K11181 only in D18 in Figure 4) in comparison to E03 and A04 which show fewer *Deltaproteobacteria*- and more *Alphaproteobacteria-*associated genes (Figure 5). We also analyzed sulfur oxidation marker *soxB* and D18 sediment is clearly different again as indicated by the absence of *Alphaproteobacteria*-associated genes. We found *sat* and *aprA* homologous sequences in all sediment samples with some differences in the number of associated homologs and the taxonomy between samples; E03 and A04 metagenomes are similar, both having a high proportion of sequences associated to *Alphaproteobacteria* and almost no sequences belonging to *Deltaproteobacteria* in contrast to D18 where there is a high amount of *Deltaproteobacteria* and no *Alphaproteobacteria-*associated genes (Supplementary Figure S1).

**FIGURE 6:**
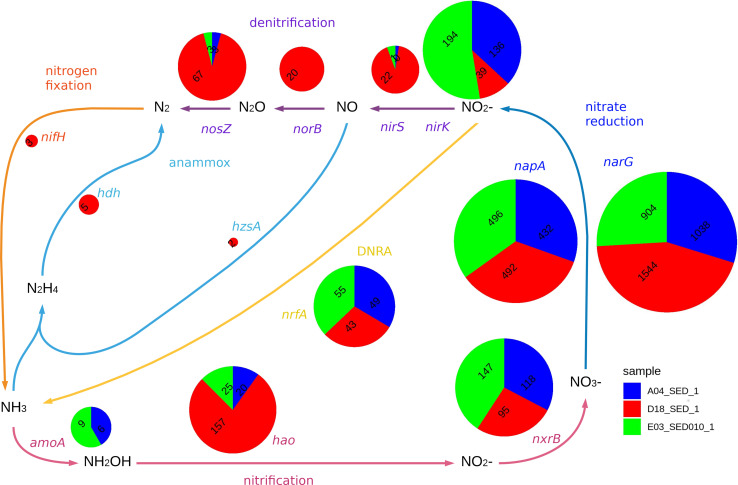
The figure displays the relative abundance of genes with homology marker genes in nitrate reduction (*narG, napA*) and related pathways (*nirS, nirK, nrfA, norB, nosZ, hzsA, hdh, amoA, hao, nxrB, nifH*). Each pie chart radius is proportional to the logarithm of the sum of relative abundances for the three samples. DNRA stands for Dissimilatory Nitrate Reduction to Ammonia. The microbial nitrogen cycle scheme was based on (Simon and Klotz, 2013). Classification for each gene is included in Supplementary Figure S2.

We constructed a graftM package using the manually curated database of Müller et al. (2015). This phylogeny can identify *dsrAB* from archaea or bacteria and also what group they correspond to reductive bacteria, oxidative bacteria and reductive archaea. Sequences related to *dsrAB* show the greatest contrast in the number of associated homologs and taxonomic groups between sediment samples. The A04 and E03 samples possess a low abundance of reads (Figure 6, top left), and all of them are related to the oxidative *Gammaproteobacteria* oxidative groups. For the D18 sediment, the majority of associated homologs correspond to the reductive clusters (89%). The biggest cluster belongs to *Deltaproteobacteria* (51%) followed by the environmental supercluster (30%), that is composed of two lineages known as sulfate reducing families with no cultivated groups (Müller et al., 2015). The most significant difference between sediments is shown in the relative abundance of *dsrAB* gene. Almost no homologs were found for A04 and E03, while in D18, most homologs correspond to the reductive type metabolism and their taxonomy shows a predominance of the Deltaproteobacteria-supercluster of *Desulfobacteraceae* (45%), *Desulfobulbaceae* (26%), *Desulfatiglans anili* lineage (16%) and *Syntrophobacteraceae* (12%) families (Figure 6, top left). Sequences related to *soxB* were found in all three sediment samples (relative abundance numbers of predicted genes for soxB in A04SED sample, 29; for E03SED sample, 15; and for D18SED sample, 19). However, sequences assigned to *Alphaproteobacteria* only appeared in A04 and E03 sediment samples (Figure 6, top right).

#### Nitrogen Metabolism

We searched for sequences homologous to genes involved in nitrogen fixation, nitrification, nitrate reduction, denitrification, DNRA, and anammox in all three sediment samples. However, we only identified homologs of anammox and nitrogen fixation pathways genes in the D18 sample (Figure 6), and conversely, *amoA* homologs (i.e., *amoA* of AOA and AOB, and *pmoA*) only in A04 and E03 sediment samples, all of them related to archaeal sequences (Supplementary Figure S2); *hao* homologs (nitrification marker) were found in the three sediment samples, although the majority were classified as distant homologs (Supplementary Figure S2). Both A04 and E03 sediments display a higher abundance of *nxrB* homologous sequences while D18 sediment samples showed more related to the *narH* gene.

We found homologous sequences to *narG* and *napA*, the typical nitrate reduction markers, and *nrfA*, a homolog of *dnrA*, in the three sediment samples. Sequences affiliated with *Deltaproteobacteria* stand out in the D18 sample. Meanwhile, sequences related to *Alpha-* and *Gammaproteobacteria* were more prevalent in samples from Perdido (A04 and E03). We found *nirK* related sequences (a denitrification marker) in all sediment samples. However, a significant proportion was present in A04 and E03. We also observed sequences related to the experimentally characterized *nirK* group only in those samples. A higher number of sequences related to *nirS* were detected in the D18 sample, and were associated to *Betaproteobacteria* and *Gammaproteobacteria*. *norB* related sequences were only found in the D18 sediment sample (from *Betaproteobacteria* and *Gammaproteobacteria*). Previous reports have shown the existence of two *nosZ* clades: TAT-dependent or typical Z-type NosZ protein, and Sec-dependent or atypical NosZ. TAT-dependent is commonly found in *Alpha, Beta* and, *Gammaproteobacteria* capable of complete denitrification, while Sec-dependent are present on a broader group of organisms with diverse nitrogen metabolism (Sanford et al., 2012; Jones et al., 2013). We only identified Sec-dependent *nosZ* in A04 and E03, however, we found both Sec-dependent and TAT-dependent homologous sequences in D18 (Supplementary Figure S2).

The gene marker for nitrogen fixation, *nifH*, was only found in D18 (*nifH* sequences related to cluster IV), and the remaining sequences could not be associated to any previously characterized *nifH* clusters. The anammox (anaerobic ammonium oxidation) process was detected only in the D18 sediment sample, with a small proportion of sequences related to *hzsA* and *hdh* of *Planctomycetes*, suggesting the possible presence of anammox bacteria in D18 sediment sample in low abundance.

#### Methanogenesis, Anaerobic Methane Oxidation and Wood–Ljungdhal Pathway

The best marker gene for methanogenesis and anaerobic oxidation of methane (AOM) is *mcrA* wich codes for the alpha subunit of methyl-coenzyme M reductase, and is used for the initial oxidation of methane or the final reduction step releasing methane in reverse methanogenesis (Friedrich, 2005). As we did not find any *mcrA* sequences, we looked for genes involved in the canonical hydrogenotrophic methanogenesis and reverse methanogenesis (Timmers et al., 2017): *fmd* (formylmethanofuran dehydrogenase), *ftr* (formylmethanofuran-tetrahydromethanopterin *N*-formyl transferase), *mch* (methenyltetrahydromethanopterin cyclo hydrolase), *mtd* (methylenetetrahydrofolate dehydrogenase), *mer* (5,10-methylenetetrahydromethanopterin reductase) and *mtr* (tetrahydromethanopterin *S*-methyltransferase).

**FIGURE 7:**
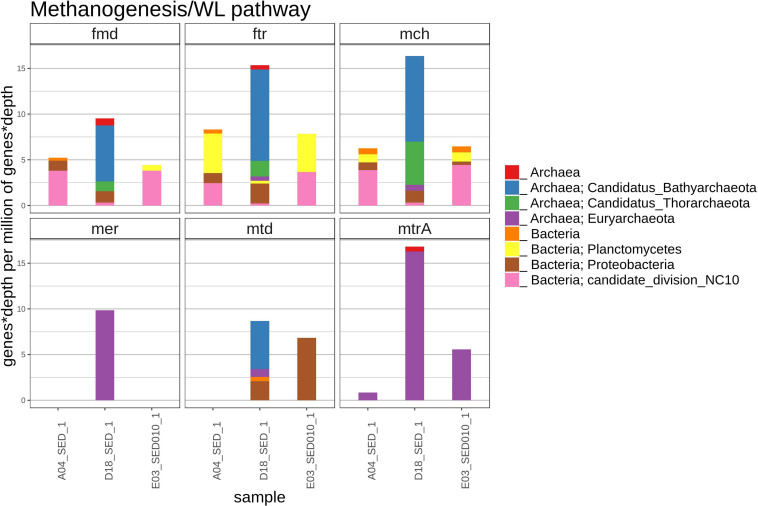
The figure shows the relative abundance (rate represented as number detected genes per million of genes in the metagenomic assembly) of genes with homology to genes *fmd* (formylmethanofuran dehydrogenase), *ftr* (formylmethanofuran:H 4 MPT formyltransferase), *mch* (methenyl-H 4 MPT cyclohydrolase), *mer* (methylene-H 4 MPT reductase), *mtd* (methylene-H 4 MPT dehydrogenase), *mtrA* (*N* 5-methyltetrahydromethanopterin: coenzyme M methyltransferase), in the methanogenesis/AOM/achaeal WL pathway in each sediment sample, and their most likely taxonomic classification according to NCBI taxonomy.

A small number of sequences from the methanogenesis/AOM pathway (*fmd*, *ftr*, *mch*, *mer, mtd*, and *mtrA*) was found in the D18 sediment sample, most of those gene sequences were related to archaea (Figure 7), which included the Bathyarchaeota and the Thorarchaeota groups, that were not found in A04 nor in E03 sediments. In the A04 and E03 samples, we found some homologous sequences to the *fmd, ftr* and *mch* genes related to candidate NC10 division bacteria and better related to the Wood–Ljungdahl pathway (WLP).

#### Aerobic and Anaerobic Hydrocarbon Degradation in Sediment and Water Samples

**FIGURE 8:**
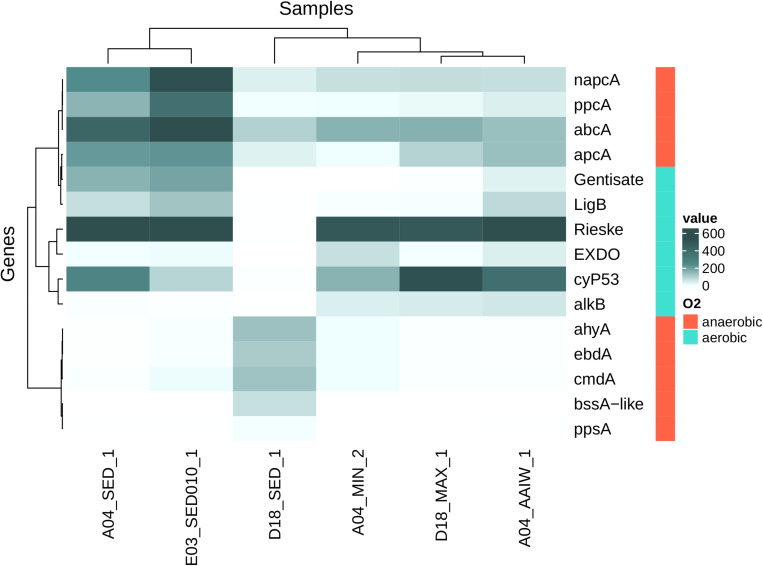
Heatmap shows homologous genes (number of genes per million of genes in metagenomic assemblies) to genes related with hydrocarbon activation step for posterior degradation. Rieske (Rieske oxygenases), cytP53 (cytochrome P53), *napcA* (naphthalene carboxylase), *abcA* (Putative Benzene Carboxylase), *ppcA* (Phenylphosphate Carboxylase), *apcA* (Acetophenone Carboxylase), LigB (protocatechuate dioxygenase), Gentisate (Cupin superfamily), *ppsA* (Phenylphosphate Synthase), *bssA-like* (Benzyl-Succinate Synthase like), *ahyA* (Alkane C2-Methylene hydroxylase), *ebdA* (Ethylbenzene Dehydrogenase), *cmdA* (*p*-Cymene Dehydrogenase), EXDO (Extradiol Dioxygenases, monocyclic bicyclic and miscellaneous substrates), *alkB* (Alkane Hydroxylase) (here gene sequencing depth was not considered). Samples and genes were sorted using hierarchical clustering.

We found sequences related to aerobic hydrocarbon degradation in the water column samples. The highest diversity of detected *alkB* homologous sequences comes from D18MAX and A04AAIW. Sediment samples A04 and E03 and A04MIN water sample showed a lower sequence diversity (Figure 8 and Supplementary Figure S3).

Sequences associated with anaerobic hydrocarbon degradation were found in the D18 sediment sample. On the other hand, we found sequences related to aerobic hydrocarbon degradation and only carboxylases related to anaerobic degradation in the A04 and E03 samples. The *bssA-like* related sequences were detected only in the D18 sediment sample. Some sequences were closely related to *assA*, but a large fraction does not associate with any of the described families from the *bssA-like* family, apparently forming new taxonomic groups (Supplementary Figure S3).

We detected sequences homologous to the Rieske oxygenase family in all but the D18 sediment samples, the detected sequences encompass almost every class in the known diversity of this family (Duarte et al., 2014). In addition, many PHDB genera were identified in all the analyzed samples. This was based on genera previously identified in the literature to be associated with metabolic pathways involved in hydrocarbon degradation and related compounds (Ramírez et al., 2020). The abundance of PHDB is evident in the sites studied. In water samples we found 34 genera reported as hydrocarbon degrading bacteria while in sediment samples 39 were present.

## Discussion

### Bacterial Community Throughout the Water Column

It is well known that *Alphaproteobacteria* are very diverse, therefore their metabolism is also very varied. However, this group tends to dominate in mesophilic environments and decrease in harsher situations; we do observe the same behavior in our water column taxonomic assignment, where *Alphaproteobacteria* decrease trough depth (Figure 3). On the other hand, we found *Gammaproteobacteria* as the most abundant class in the aphotic layer. This group of bacteria plays an important ecological role in xenobiotic degradation, as the majority of the obligate hydrocarbon degrader bacteria belong to this class such as *Alcanivorax, Alteromonas, Cycloclasticus*, and *Pseudomonas* (Yakimov et al., 2007). All of them were identified in the aphotic layers with an average abundance range of 0.01–5.0%. We observed a particular enrichment of *Alcanivorax* 23%, *Alteromonas 26.8*%, and *Vibrio* 64% in A04 DEEP, D18 MIN and C10 DEEP respectively. These findings suggest that bacteria of the water column are actively degrading hydrocarbons, especially those from the higher depths, perhaps maintaining the equilibrium of the GoM hydrocarbon levels.

### Bacterial Community Throughout Sediments

Anthropogenic inputs are detected in both NW and SE regions, increasing organic matter inputs. It has previously been reported that the presence of diverse hydrocarbons in NW GoM is due to various factors, especially to the organic material discharged by the Laguna Madre, Bravo, Soto La Marina and Pánuco rivers, which contribute to the complex mixture of organic compounds that accumulate in the marine sediments (Macdonald et al., 2015; Aguilera et al., 2019). Bacterial taxonomic profiles only showed significant differences for deep and shallow sediments and it was not possible to see the differences between NW and SE regions that the whole-metagenome data showed. However, we are aware that D18 sample is not enough to characterize a complete region, and even more after the variations that we have seen in SE GoM (Godoy-Lozano et al., 2018), where we identified changes in the bacterial community possibly due to perturbations generated by anthropogenic oil activities. Sanchez-Soto et al. (2018), reported a similar bacterial community from the Perdido Fold Belt as in this work. In both reports we observe several coincidences at the Class, Order, and even at Genus level. However, some of the taxonomic assignments vary due to the use of different databases.

### Linking Taxonomy and Metabolism

Due to the technical and amplification biases of amplicon and shotgun metagenomics, we do not expect a correlation between the taxonomic results from both methods, therefore, we report amplicon data for our taxonomy analysis and we use WMS data for networks and metabolism. Networks could be built as WMS gave us a closest hit for each sequence for both genus and functional gene. However, we are aware that in fact each genus and each gene represent a whole group of similar bacteria or genes. Heterogeneity values determined in the networks show that sediments present a more complex community than water (Figure 4), suggesting a complex coordination-like dynamic drives the population in this habitat, as indicated in a recent network interpretation study (Pinheiro and Hartmann, 2017). In contrast, traits are more independent in the water column resulting in a less functionally redundant system, with specialized bacteria as *Synechococcus* and *Prochlorococcus* in the MAX layer. The recurrent presence of *Acinetobacter* and *Pseudomonas* in all layers as taxonomical keystones, and K01563 and K02567 as metabolic hubs, supports the presumption that bacteria adapted to hydrocarbons and xenobiotics are dominant in these sites. Nevertheless, networks also reflect the nitrogen, sulfur, and carbon pathways that are central in the biogeochemical cycles, and the functional redundancy that exists in sediments.

### Marker Genes for Anaerobic Mineralization of Organic Matter

Marine sediments can be divided into three zones from top to bottom: oxic, suboxic, and reduced. Aerobic respiration and nitrification take place in the oxic zone, sulfate reduction and denitrification occur in the anoxic zone. Dissimilatory sulfate reduction takes place in the top part of the reduced zone, and below all, carbon dioxide reduction (methanogenesis) is carried out. The potential for denitrification solely found in A04 and E03 suggests that our samples of the 10 centimeters below the surface (cmbs) only reached the oxic and suboxic zones; this is consistent for deep samples, where we expect deeper oxygen penetration depths (Kristensen, 2000). On the other hand, in the same 10 cmbs of the D18 sediment sample, dissimilatory sulfate reduction potential and denitrification stand out, which along with the presence of *hao* and absence of *amoA*, suggest a thin oxic zone above the anoxic zone, as would be expected for shallower sediment samples (Kristensen, 2000). It would seem that natural hydrocarbon emanations could be playing an important role as accessible organic matter that depletes the oxygen rapidly, although we cannot yet rule out the involvement of other sources of organic matter such as river’s sewage, dead fauna or fecal pellets.

### Sulfur Metabolisms (DSR and Sulfur Oxidation)

The *dsrAB* gene has been previously detected in *Alphaproteobacteria, Betaproteobacteria, Gammaproteobacteria, Deltaproteobacteria*, and *Chlorobi* using reverse dissimilatory sulfite reduction pathway (RDSR) (Loy et al., 2009; Swan et al., 2011; Sheik et al., 2014; Callbeck et al., 2018; Koch and Dahl, 2018). The homologous genes related to dissimilatory sulfate reduction (*sat, aprA*, and *dsrAB*) suggest the presence of a sulfate reduction population in the D18 sediment associated to *Deltaproteobacteria*; on the other hand, homologs of the same genes and of *soxB* suggest the presence of *Alphaproteobacteria* sulfur oxidizing populations in the A04 and E03 sediment samples.

The relative abundance of *sat* and *aprA* homologous genes is similar; however, their associated taxonomy has a main difference, in D18 sediments seem to be coming from a *Deltaproteobacteria* population and in A04 and E03 from the *Alphaproteobacteria* group (Supplementary Figure S1). The bacterial community in D18 sediments has the typical *Deltaproteobacteria* members of anoxic marine sediments shown to have an important role in acetate assimilation and carbon cycling (Jørgensen, 1982). These communities appear to be predominantly composed by *Desulfobulbaceae*, *Desulfobacteraceae*, and *Syntrophobacteraceae* (Figure 6). The latest have a diverse metabolism that allows them to grow with or without sulfate (Kuever, 2014; Wörner and Pester, 2019). *Desulfobulbaceae* also appears to have an essential role in acetate consumption, while *Desulfobacteraceae* remains dominant in the two groups (Dyksma et al., 2018). Interestingly, organisms from these families have been previously shown to anaerobically degrade hydrocarbons (Stagars et al., 2016; Davidova et al., 2018), to carry out acetate assimilation (Dyksma et al., 2018) and to be syntrophic partners of methanogenic and methanotrophic archaea (Mcglynn et al., 2015; Oberding and Gieg, 2016). A new yet unresolved pathway for sulfate reduction has been proposed for a syntrophic microbial consortium that mediated the AOM coupled to sulfate reduction and polysulfide disproportionation (Milucka et al., 2012). Remarkably, sulfate was reduced by an unknown mechanism in the archaeal partner resulting in the formation of disulfide and not by the deltaproteobacterial partner that harbors the canonical DsrAB-based pathway (Müller et al., 2015).

The dominance of the *Alphaproteobacteria* population increases within the *soxB* sulfur oxidation marker gene in the A04 and E03 sediments, followed by *Epsilonproteobacteria* and *Gammaproteobacteria*, all these groups have been reported to have species related to sulfur oxidation bacteria (SOB) (Yamamoto and Takai, 2011). Sediments from D18 show an absence of *Alphaproteobacteria*, and only *Gamma* and *Epsilon* were found with this marker gene. Marine microorganisms from *Epsilonproteobacteria* class are known to use the SOX system for sulfur oxidation, and *Gammaproteobacteria* use the reverse sulfate reduction (RDSR) apart from the sox system (Yamamoto and Takai, 2011), it has been suggested that they constitute 40–70% of the autotrophic sulfur oxidizers (Lenk et al., 2011; Boschker et al., 2014; Jørgensen et al., 2019). The high abundance of *Alphaproteobacteria* in the sediments from A04 and E03 is uncommon as there are few reports of them being the dominating group of sulfur oxidizers. Further studies are required to determine if the abundance of this group is due to the type of sediment and associated with abiotic variables.

### Methane Metabolism and Wood–Ljungdahl Pathway (WLP)

Methane metabolism in the ocean is of particular interest because of its global warming potential, 23 times that of carbon dioxide (Brown et al., 2014). Also oceans act as a sink for atmospheric methane, and contribute to global methane emissions by 1–4% (Karl et al., 2008). We decided to look for methane metabolism genes in our sediments, given the importance of methane and the numerous oil and gas seeps of diverse origin in the GoM (Kennicutt, 2017). Methane related metagenomic sequences represent a small proportion of the total sequences, we do not rule out the possibility of *mcrA* absence due to incomplete sequencing. With respect to the other explored genes, we could detect only methanogenesis genes in D18 sediment and some carbon fixation genes of the WLP in E03 and A04 sediments. The relation between WLP and methanogenesis is considered to be an important and ancient energy generation and carbon fixation metabolism, where these two metabolisms share some homology and gene conservation between archaea and bacteria (Borrel et al., 2016).

The presence of anaerobic archaeal communities with energetic coupling types that have not yet been completely explored, where reducing power comes from oxidation of organic compounds using enzymes related to WLP and methanogenic pathways is likely in our D18 sediment sample. All of the identified methanogenic organisms belong exclusively to three phyla of the archaeal domain: the well-known Euryarchaeota (Methanomicrobiales), the recently discovered Bathyarchaeota (former Crenoarchaeota group) (Evans et al., 2015) and the very recent Verstraetearchaeota phylum (Berghuis et al., 2019). We detected the known WLP genes carried by NC10 bacteria and Planctomycetia (Chistoserdova et al., 2004; Adam et al., 2019; Kalyuzhnaya et al., 2019). WLP is commonly found in acetogenic bacteria, archaea and recently has been found in non-methanogenic and non-methylotrophic archaea and bacteria, where its function remains unclear in the absence of methyl-coenzyme M reductase complex (MCR) and *N5*-Methyltetrahydromethanopterin:coenzyme M methyltransferase complex (MTR) complexes (Borrel et al., 2016; Chistoserdova, 2016); it has been suggested that it could be involved in other processes than methanogenesis like extra H2 production during fermentation or formaldehyde detoxification (Adam et al., 2019). Recently, methanogenesis has been linked to alkane degradation through a divergent MCR within some Euryarchaeota capable of degrading ethane (Chen et al., 2019; Laso-Pérez et al., 2019; Wang et al., 2020), which opens the possibility of having methanogens degrading hydrocarbons with MCRs not detected by this study.

### Dissimilatory Nitrate Reduction and Adjacent Pathways Related to the Nitrogen Cycle

Dissimilatory nitrate reduction is a key step in the nitrogen cycle, as nitrate is the second preferred electron acceptor after oxygen, useful for metabolism in anaerobic conditions (Hensen et al., 2006). We identified nitrate reduction potential in the three sediment samples, having a high relative abundance and diversity of both nitrate reductases we searched for, this is also consistent with the wide distribution of nitrate reduction in nature (Kraft et al., 2011).

Nitrogen in nitrite produced by dissimilatory nitrate reduction can go back to ammonium (DNRA) or escape as dinitrogen gas (denitrification or anammox), which depends on the environmental conditions and the genomic composition of the community (Kraft et al., 2011). These enzymatic steps can also be coupled to electron transport and energy conservation processes (Simon and Klotz, 2013). The abundance of *nrfA* homologs (gene marker for DNRA) indicates the potential to return nitrite into ammonium in all samples. Nitrite reductases that catalyze the initial step for nitrogen loss (*nirK* and *nirS*) are also present in the three samples, notably *nirS* is more abundant in D18 and *nirK* dominates in A04 and E03. The abundance patterns and associated populations for *norB* and *nosZ*, suggest a complete denitrification potential in the D18 sample. Nitrate reduction generally occurs when oxygen is depleted in the environment. The presence of *nirS, norB, nosZ* (denitrification pathway) in D18 sediment, complements the findings that this sediment is more anoxic.

We found the marker genes for anammox only in the D18 sample, and in low abundances. So far, the proposed pathways for anammox start with nitrite reduction into nitric oxide by the *nirS* enzyme (Kartal et al., 2011). Annammox has been exclusively found in five genera belonging to the phylum *Planctomycetes* (Kartal and Keltjens, 2016) but no sequence found here could be directly associated with any of those five genera. Also, the *nifH* homologous sequences we found were not related to any characterized nitrogen fixing clusters (Chien and Zinder, 1994; Dang et al., 2009).

Nitrification transforms ammonium into nitrate in the presence of oxygen (Kuypers et al., 2018). The *amoA* (ammonia monooxygenase subunit A) and *hao* (hydroxylamine oxidoreductase) genes have been previously used to assess the environmental diversity of aerobic ammonium oxidizing microorganisms (Rotthauwe et al., 1997; Schmid et al., 2008; Alves et al., 2018). We only detected *amoA* related sequences in A04 and E03, suggesting that a community of aerobic archaea performing ammonium oxidation is present only there and not in D18.

Homologous sequences to the *hao* and *nxrB* genes are present in the three sediment samples, however, their homology to other genes in the nitrogen cycle hinders the direct interpretation of the results for these two steps, for example a large portion of *nxrB* homologs in D18 sediment sample is more similar to respiratory nitrate reductase (*narH)*, that encodes a subunit of the NAR complex involved in nitrate reduction (Simon and Klotz, 2013).

An accurate interpretation is hard to achieve, as the nitrogen cycle and the enzymes involved are a complex system, as Simon and Klotz (2013) have analyzed. The role enzymes play depends upon both environmental conditions and the organism in question. Many enzymes show homology between different steps in the cycle, some enzymes display promiscuous activity, and most enzymes belong to protein complexes. Therefore, the query enzymes we are taking here, give us only an overview of the metabolic potential for nitrate reduction and related steps.

### Hydrocarbon Degradation in Sediment and Water Samples

We expected the presence of diverse sequences related to hydrocarbon degradation as previous studies in the water and sediments in the GoM have shown the presence of PHDB (Godoy-Lozano et al., 2018; Moreno-Ulloa et al., 2019; Ramírez et al., 2020). It is important to note that some of the most abundant bacterial genera reported in this work are related to the biodegradation of contaminants and support the idea that microbial communities in deep-sea sediments of GoM have the potential to metabolize a variety of organic compounds, including oil, crude oil and its derivatives.

We found sequences associated to anaerobic hydrocarbon degradation in the D18 sediment sample, sequences related to both anaerobic and aerobic degradation in A04 and E03. Finally, water samples contain primarily sequences related to aerobic degradation. The diversity of sequences suggests microorganisms have the intrinsic potential to degrade a wide range of hydrocarbon compounds, as might be the case in an oil spill event. *alkB* genes are associated with the degradation of C10-C28 alkanes (Van Beilen and Funhoff, 2007; Nie et al., 2014; Muriel-Millán et al., 2019), and we detected the majority of these sequences in water samples. The high diversity of Rieske oxygenases in water samples and in the A04 and E03 sediments, indicates there is potential for degradation of diverse aromatic compounds such as benzoate, benzene, toluene, phthalate, naphthalene or biphenyl (Duarte et al., 2014).

The AlkB and Rieske oxygenases enzymes are active under aerobic conditions, but hydrocarbon degradation can also occur under anaerobic conditions. The D18 sediment sample shows a high diversity of *bssA-like* sequences, which are involved in hydrocarbon (both aromatic and aliphatic compounds) anaerobic degradation activation step through fumarate addition (Acosta-González et al., 2013) and have been reported in sulfate reducing bacteria and nitrate reducing bacteria (Kropp et al., 2000; Rabus et al., 2001). Other studies have reported the presence of *bssA*-like sequences in bacterial consortia capable of growing using hydrocarbon compounds under anaerobic conditions (Callaghan et al., 2010).

## Conclusion

The microbial diversity and structure of the bacterial communities in water samples and surface sediments of the NW and SE region of the GoM revealed differences between these two geographical zones particularly in sediments. The high relative abundance of sulfate reduction, denitrification marker-genes and a high numbers of anaerobic genes and bacterial genera shown in networks and also in our drawn metabolic pathways leads us to conclude that our SE GoM sample is a more oxygen depleted sediment with the presence of an anaerobic bacterial community, explained perhaps by the high organic matter deposits in seafloor coming from both, river discharge and hydrocarbons. The abundance of putative hydrocarbon degrading bacteria and hydrocarbon degradation genes in both studied areas could be indicative of impacted zones caused by the presence of hydrocarbons in the environment that could influence the equilibrium of biogeochemical cycles. Our findings contribute to understanding the role of indigenous GoM bacterial taxa in biogeochemical cycles and hydrocarbon-degrading metabolism that would have a potential role in bioremediation strategies for oil-spills in water and marine sediments.

## Data Availability Statement

The datasets presented in this study can be found in online repositories. The names of the repository/repositories and accession number(s) can be found in the article/ Supplementary Material.

## Author Contributions

KJ and LS conceived and designed the experiments. LR, FG-G, AM-S, EG-L, and AE-Z analyzed and interpreted the data. LR, FG-G, AM-S, KJ, and LS wrote the manuscript and prepared the figures and tables. AL-N managed the L4-CIGOM consortium resources and guaranteed their availability to perform the experiments and analysis. LP-L coordinated the IBT-L4-CIGOM group. EM, RG-R, AL, AS-F, and LP-L contributed to the improvement of the project and reviewed the final version of the manuscript. All the authors contributed to the article and approved the submitted version.

## Conflict of Interest

The authors declare that the research was conducted in the absence of any commercial or financial relationships that could be construed as a potential conflict of interest.

## Footnotes

1 cigom.org
