# Supplementary material for: Metagenomic Profiling and Microbial Metabolic Potential of Perdido Fold Belt (NW) and Campeche Knolls (SE) in the Gulf of Mexico
Source: Front Microbiol. 2020 Aug 11;11:1825. doi: 10.3389/fmicb.2020.01825 (PMC7438803; doi:10.3389/fmicb.2020.01825)
Supplement: Supplementary file 4 [file Data_Sheet_1.PDF]

frontiers

Supplementary Material

1 SUPPLEMENTARY DATA

1.1 gpkg files

The gpkg files built in this study can be found at *github.com/garciafertson/mmf1_gpkgs*

1.2 network files

The *Supplemetary_network_files.zip* file contains a folder with cytoscape CYS network files constructed as explained in **Materials and Methods section**. Ovals represent Genus and squares represent KOs. An edge represents if a KO-genus pair is present, and edge width is proportional to the log2 of total counts. Methane metabolism is shown in light gray and methanogenesis in gray; sulfur oxidation in ocre, sulfate reduction in yellow and sulfur uptake/assimilation in lime; nitrate reduction/denitrification in sky blue and nitrogen metabolism in blue; alkanes/hydrocarbon degradation in dark brown, aromatics degradation in brown and chlorinated compound degradation in purple; oxygenic photosynthesis in green and aerobic respiration in pink. File names ending with *min10.cys* display only edges with a total count above 10. File names ending with *complete.cys* display edges with at least one count.

2 SUPPLEMENTARY TABLES AND FIGURES

2.1 Tables

Tables are included as separate XLSX files.

Supplementary_Table_1 CTD_DATA. **water_column sheet)** CTD data statistics by water column level **region sheet)** CTD data statistics by region.

Supplementary_Table_2 Sample stats. **WMS sheet)** Sequencing and assembly statistics for Whole Metagenomic Shotgun data. **Amplicon sheet)** Sequencing statistics and alpha diversity for Amplicon, OTU analysis. **ASVs Amplicon sheet)** Alpha diversity for Amplicon, ASV analysis.

Supplementary_Table_3 KO numbers. **Searched KO list sheet)** Selected gene list (as KO numbers) displayed in networks. **Key genes and genus found sheet)** Key KO numbers and genera found in networks by water column level.

2.2 Figures

Figures are also included as separate PNG files.

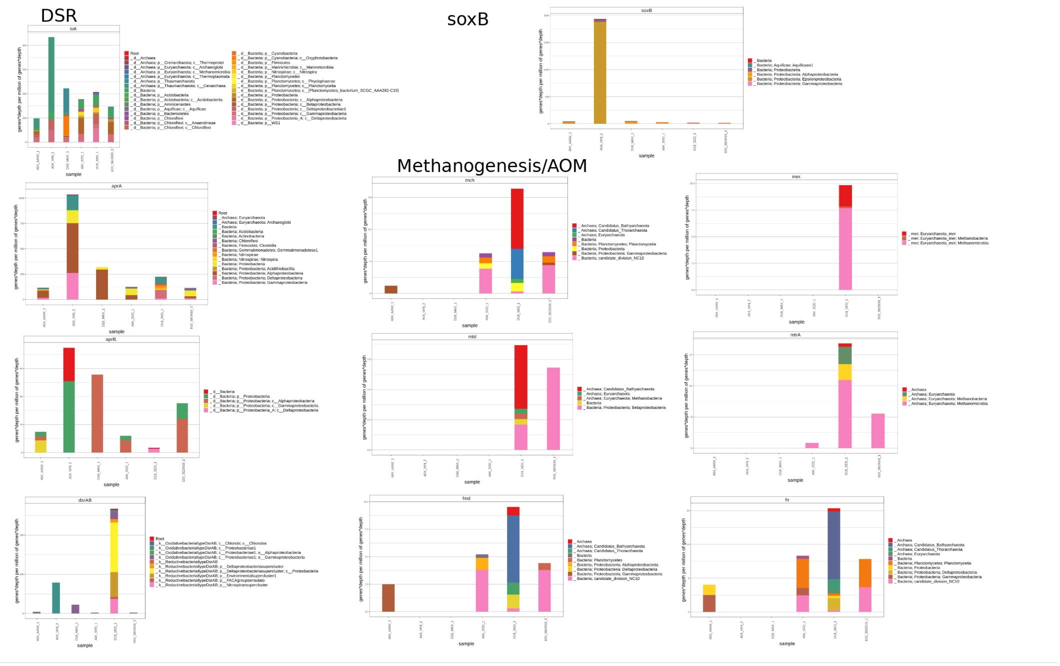

Figure S1. The bar plots show the relative abundance and most likely taxonomic assignation using graftM packages for genes related to **(DSR)** Dissimilatory Sulfite reduction: *sat, aprA, dsrAB*, **(soxB)** Sulfate oxidation: *soxB* and **(Methanogenesis/AOM)** *mcrA, mer, fmd, ftr, mch, mtd, mtrA*

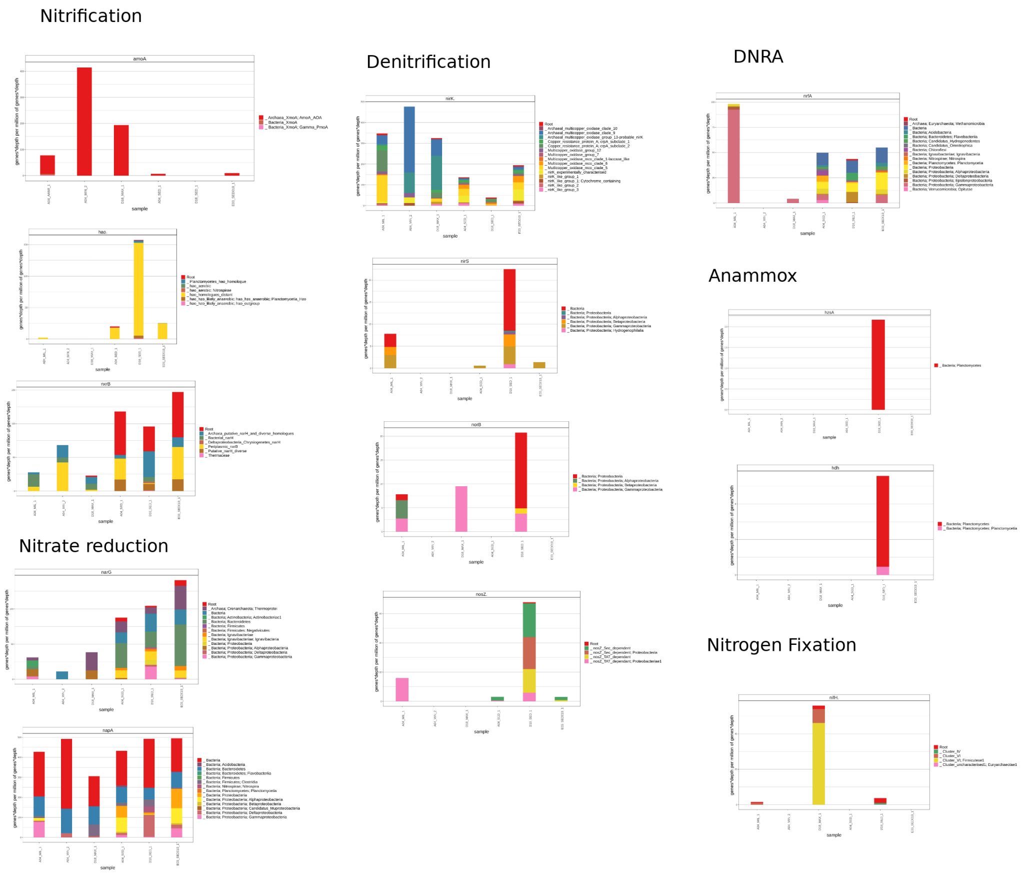

Figure S2. The bar plots show the relative abundance and most likely taxonomic assignation using graftM packages for genes related to **(Nitrification)** *amoA, hao, nxrB*, **(Nitrate reduction)** *narG, napA*, **(Denitrification)** *nirS, nirK, nosZ, norB*, **(DNRA)** Dissimilatory Nitrate Reduction to Ammonia: *nrfA* **(Anammox)** *hzsA, hdh, hao and* **(Nitrogen fixation)** *nifH*.

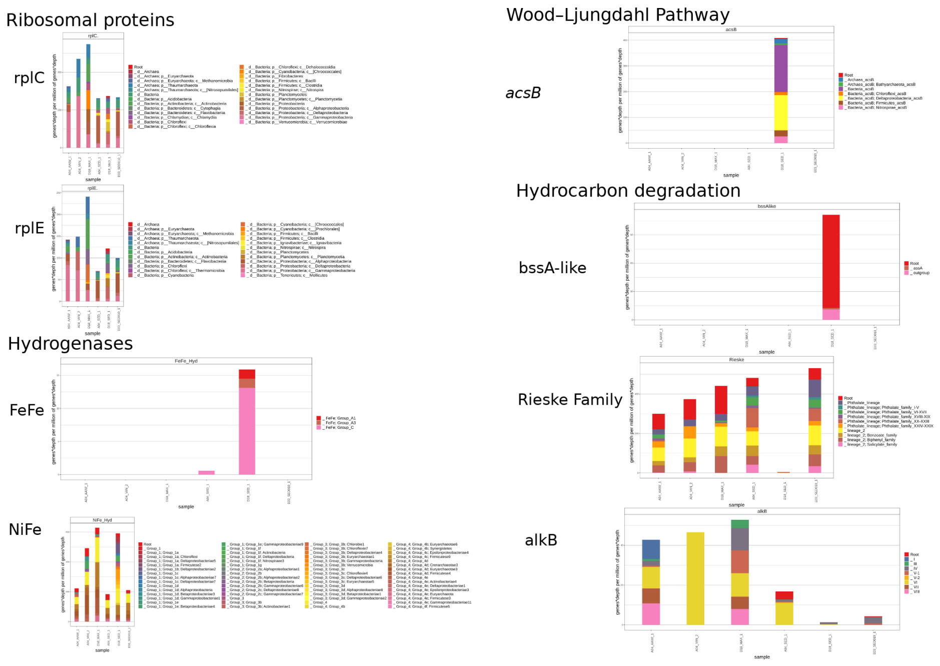

Figure S3. The bar plots show the relative abundance and most likely taxonomic assignation using graftM packages for genes related to **(Ribosomal Proteins)** *rplC, rplE*, **(Hydrogenases)** Fe-Fe Hydrogenases, Ni-Fe Hydrogenases, **(Wood-Ljungdahl Pathway)** *acsB*, **(Hydrocarbon degradation)** *alkB, bssA-like*, Rieske superfamily genes
